# Effect of adjuvanted and standard sublingual immunotherapy on respiratory function in pure rhinitis due to house dust mite over a 5-year period

**DOI:** 10.1186/s40413-016-0132-1

**Published:** 2017-02-14

**Authors:** Maurizio Marogna, Alessandro Massolo, Giovanni Passalacqua

**Affiliations:** 1 Pneumology Unit, Cuasso al Monte, Macchi Hospital Foundation, Varese, Italy; 2 Department of Ecosystem and Public Health, Faculty of Veterinary Medicine, University of Calgary, Alberta, Canada; 3 O’Brien Institute for Public Health, University of Calgary, Alberta, Canada; 4 Department of Internal Medicine, Allergy and Respiratory Diseases, IRCCS San Martino-IST-University of Genoa, Pad. Maragliano, Ospedale San Martino, L.go R. Benzi 10, 16133 Genova, Italy

**Keywords:** Sublingual immunotherapy, Adjuvants, House dust mite, Allergic rhinitis, Allergic asthma

## Abstract

**Background:**

Allergen-specific immunotherapy (AIT) still remains the only causal treatment for IgE mediated respiratory diseases (rhinitis/asthma) In addition to the observed clinical decrease in symptoms, AIT can provide a long-lasting and preventive effect. In particular it can modify the progression from rhinitis to asthma.

**Methods:**

The study was observational, open, non randomized, controlled, prospective and performed in a real-life setting. Patients with pure mite-induced allergic rhinitis were followed-up, receiving adjuvanted SLIT (aSLIT), standard SLIT (sSLIT) or drug treatment alone, according to their preference starting between 2008 and 2009. The possible onset of asthma, changes in pulmonary function and bronchial hyperreactivity (BHR) were assessed over a 5-year horizon. Also the onset of new sensitizations and symptoms-medication score (SMS) were evaluated.

**Results:**

One hundred forty two patients fulfilling the inclusion criteria were assessed at baseline, and 124 had the 5-year evaluation (age range 8–57, 69 male). After 5 years of treatment, new sensitizations appeared differentially among treatments with 58.1% of new sensitizations in the drug treatment group, 13.2% in the sSLIT patients, and 8.1% in the aSLIT patients. At the end of 5 years, SMS significantly changed (*P* < 0.001) in all groups, with a negative trend for controls, as compared to the SLIT treatments. The SMS decreased in both SLIT groups at 5 years, with no change in patients on drug treatment alone. The use of salbutamol (absent at baseline), showed an overall increase only in the group receiving drugs alone with a significant difference at 5 years (*P* < 0.001). Considering the MCh challenge, there was a difference among treatments (*P* < 0.001) in PD20 after 5 years: the control group had a lower PD20 at 5 years. No significant difference in PD20 was detected between sSLIT and aSLIT. The FEV1 significantly decreased in controls, with no change in the sSLIT group and a significant increase in aSLIT as compared to sSLIT.

**Discussion:**

Despite the limitations inherent to a real-life setting study (absence of randomization and control, small sample size, lack of intermediate timepoint assessment) the results of this study evidenced that the investigated SLIT product, either adjuvanted or not, had a positive effect on the evolution of respiratory allergy due to house dust mite.

**Conclusion:**

In the real life setting, considering a 5-year period, aSLIT and sSLIT reduced the onset of new sensistizations and maintained intact the pulmonary function, as compared to patients receiving drug treatment alone.

## Background

Allergen-specific immunotherapy (AIT) still remains the only causal treatment for IgE mediated diseases, namely respiratory (rhinitis/asthma) and hymenoptera venom allergy [1–3]. The clinical effects of AIT relies on its complex mechanisms of action, involving the cellular and humoral response to the responsible antigens [4]. AIT reduces the severity of IgE mediated reactions and the subsequent inflammation, by redirecting the T-cell responses, increasing the secretion of regulatory cytokines, and the synthesis of allergen specific IgG4 (that blocks the IgE facilitated antigen presentation) [4, 5]. In addition to the observed clinical decrease in symptoms, AIT can provide a long-lasting and preventive effect [6]. In particular it can modify the progression from rhinitis to asthma. Currently, both the subcutaneous (SCIT) and the sublingual (SLIT) routes are validated and accepted for clinical use. In parallel, several improvements have been proposed, including new routes of administration, recombinant molecules and adjuvants [7]. Adjuvants, in particular, are expected to enhance the antigenic effect of the allergen, reinforcing the skew towards the TH1 phenotype. Bacterial wall-derived adjuvants, that engage the toll-like receptors 9 (TLR-9) [8], have displayed an additional clinical effect in clinical trials, and in fact commercial products are now available. This effect has been recently applied also to SLIT, but the long-lasting and preventive effects have never been studied in the real-life setting.

In this prospective, open, real-life setting observational study we assessed the disease modifying effect (changes in respiratory function, onset of bronchial hyperreactivity, onset of new sensitizations) in patients monosensitized to dust mites and with pure allergic rhinitis over a 5-year period, receiving both natural or adjuvanted SLIT.

## Methods

The study was observational, open, prospective, performed in a real-life setting and therefore not randomized. Three groups of patients with mite-induced allergic rhinitis (AR) were followed-up, receiving adjuvanted SLIT (aSLIT), standard SLIT (sSLIT) or drug treatment alone, starting between 2008 and 2009. The possible onset of asthma, changes in pulmonary function and bronchial hyperreactivity (BHR) were assessed over a 5-year horizon. Also the onset of new sensitizations was evaluated. The observational study was performed at the Ospedale di Cuasso al Monte- Azienda Ospedaliera Fondazione Macchi-Varese, Italy. Since the study used the standard of care, no Ethical Committee evaluation was required. All patients signed an informed consent for the treatment of personal data, as per usual procedure.

We evaluated consecutive patients monosensitized to house dust mite (HDM), confirmed by positive skin prick test and/or CAP-RAST assay, and suffering from moderate/severe persistent allergic rhinitis. None had asthma [9, 10], as documented by clinical history, pulmonary function test (PFT) and methacholine (MCh) challenge. Skin prick test were performed using a commercial panel of extracts (Alk Abellò, Lainate, Milan, Italy), including HDM, grass, birch, parietaria, cypress, ragweed, alternaria, cat and dog dander. The results were read as per recommendations. Patients had to have a forced expiratory volume in 1 s (FEV1) > 80% of predicted, and a negative MCh challenge that is a provocative dose causing a 20% fall in FEV1 (PD20) >1290 mcg. Patients with malignancies, autoimmune diseases or immune-deficiencies nasal mechanical abnormalities (septal deviation, polyps), cardiovascular or psychiatric disorders, pregnancy, were considered not eligible for AIT [1–3]. After the diagnosis of allergic rhinitis was established (years 2008–2009), SLIT was proposed to patients as per guidelines. They could chose to start or not the SLIT course, administered as drops. According to their personal decision, patients received the aSLIT (Anallergo, Florence, Italy) or the standard (not adjuvanted) sSLIT (Anallergo, Florence, Italy) or drug treatment alone. All patients received cetirizine 10 mg (1 tablet/day) and budesonide nasal spray (100 mcg/nostril twice daily). Also, inhaled salbutamol (100 mcg 2–4 actuations) was prescribed in the case of unexpected asthma episodes. The SLIT course lasted 5 years, and parameters (diary card, PFT, MCh, skin test) were therefore assessed at baseline and after 5 years. The clinical diary was recorded from November to February (expected maximal exposure to mites) at baseline and after 5 years. Rhinorrhea, itching, sneezing, obstruction, conjunctival itching, tearing, wheezing and cough were graded from 0 (absent) to 3 (troublesome). Each dose of the prescribed medications was scored 1. The monthly mean of symptoms + medications score (SMS) was used for statistics. PFT and MCh challenge were carried out according to guidelines [11], by a computerized spirometer and dosimeter (Masterlab Yaeger, Wurtzburg, Germany).

Pearson Chi-square was used to compare gender distribution, drop-off and new sensitization frequencies across groups, whereas ANOVA was used to initially test differences of means among different treatments at baseline. Normality was tested using Kolmogorov Smirnov test, whereas the homoscedasticity was tested using the Welch’ test. The effects of different treatments after 5 years were tested using a Generalized Linear Model for repeated measures to compare within and between factors effects. Post Hoc tests were conducted using a Sidak correction for multiple comparisons [12]. When variability comparisons were needed, interquartile distances were computed by using the Tukey’s Hinges. Probability levels for Pearson Chi-square and Kolmogorov-Smirnov tests were computed using a complete randomisation method [13].

## Results

**Fig. 1:**
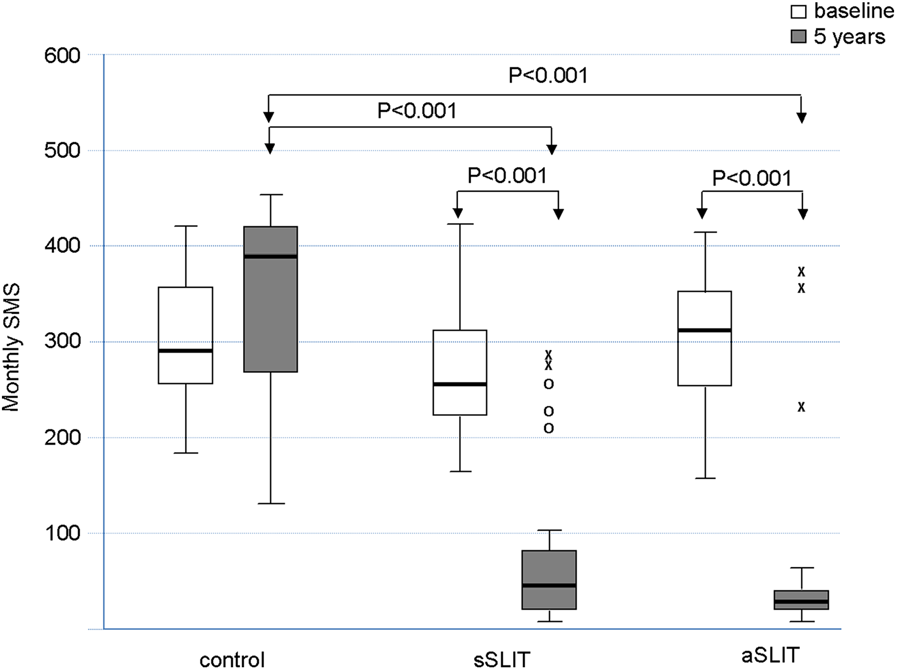
Changes in mean monthly symptoms scores (SMS) in the 3 groups of patients at baseline and after treatment. *Boxes* represent the interquartile range, *thick line* represents the 2^nd^ quartile (median); *whiskers* represent extreme values; outliers are represented as *empty dots* (more than 1.5 times the interquartile distance) or as *asterisks* (above 3 times the interquartile range). *P* values indicated above boxes

**Fig. 2:**
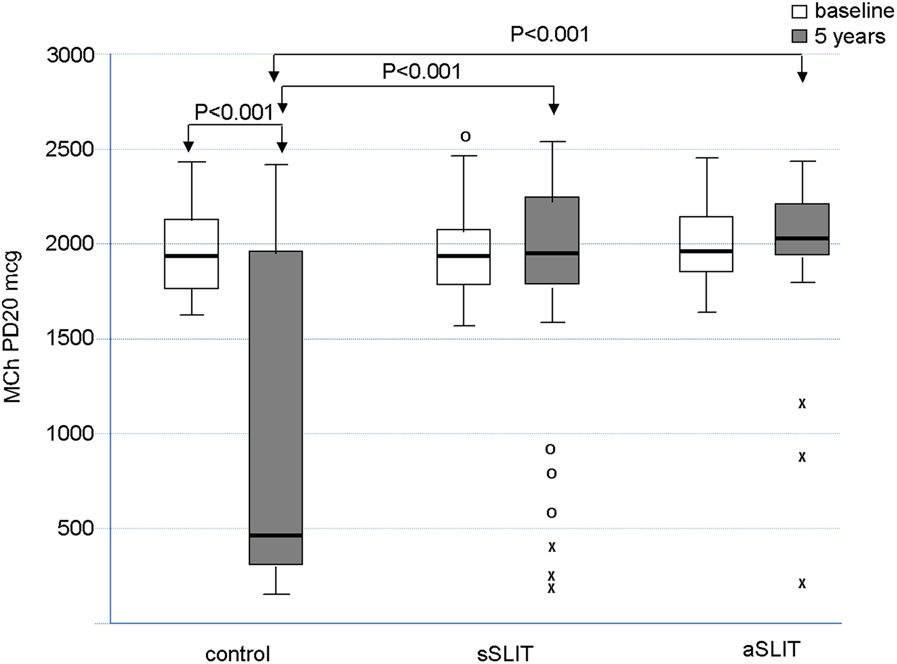
Changes in MCh PD20. in the 3 groups of patients at baseline and after treatment. *Boxes* represent the interquartile range, *thick line* represents the 2^nd^ quartile (median); *whiskers* represent extreme values; outliers are represented as *empty dots* (more than 1.5 times the interquartile distance) or as *asterisks* (above 3 times the interquartile range). *P* values indicated above boxes

**Fig. 3:**
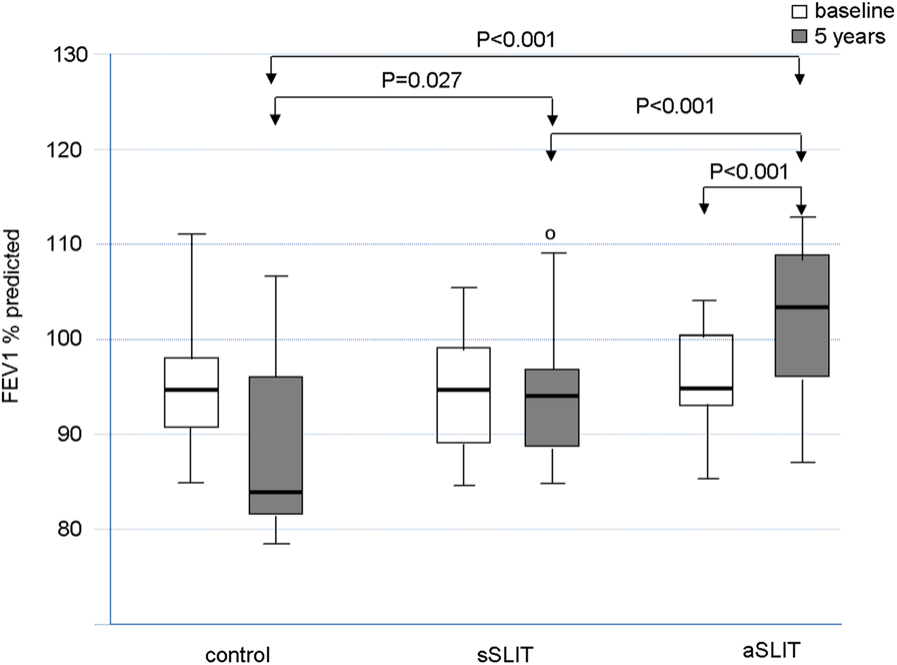
Changes in FEV1. in the 3 groups of patients at baseline and after treatment. *Boxes* represent the interquartile range, *thick line* represents the 2^nd^ quartile (median); *whiskers* represent extreme values; outliers are represented as *empty dots* (more than 1.5 times the interquartile distance) or as *asterisks* (above 3 times the interquartile range). *P* values indicated above boxes

One hundred and forty two patients fulfilling the inclusion criteria were assessed at baseline, and 124 had the 5-year evaluation (mean age 27, age range 8–57, 69 male). No significant difference was detected in baseline values for gender (*X* ^*2*^ = 0.140, df = 2, *P*_*Exact*_ = 0.948) or age (*F* = 0.400, *P* = 0.671) (Table 1). At the end of the observation period of 5 years (completed SLIT course), there were 9 drop-out in the drug treatment group (22.5%), 5 in the sSLIT (11.5%) and 4 (9.7%) in the aSLIT group, with no statistical difference (*X* ^*2*^ = 3.092, df = 2, *P*_*Exact*_ = 0.219). No age effect was detected for any of the examined parameters (GLM *P* > 0.05). After 5 years of treatment, new sensitizations appeared differentially among treatments (*X* ^*2*^ = 20.815, df = 2, *P*_*Exact*_ < 0.001), with 58.1% (18/31) of new sensitizations in the drug treatment group, in 13.2% (5/38) in the sSLIT patients, and in 8.1% (3/37) in the aSLIT patients (Table 2). The majority of new sensitizations were due to grasses and birch, as expected in this geographic area. At the end of 5 years, SMS significantly changed (*F* = 17.922, *P* < 0.001) in all groups, but with a negative trend for controls, as compared to the SLIT treatments (*F* = 66.916, *P* < 0.001). The SMS decreased in both SLIT groups at 5 years, with no change in patients on drug treatment alone (Fig. 1). Across the groups, an overall highly significant difference among treatments was detectable (*F* = 61.600, *P* <0.001), mostly due to the fact that the patients receiving drugs alone had more symptoms than the two SLIT groups (Post Hoc Sidak correction: drugs vs. sSLIT, mean difference 142.3, *P* < 0.001; drugs vs. aSLIT, mean difference 147.4, *P* < 0.001). There was no significant difference between sSLIT and aSLIT. Of note, the use of salbutamol (absent at baseline), showed an overall increased consumption only in the group receiving drugs alone (Table 3), with a significant difference in 2014 (*F*_*2,105*_ = 18.705, *P* < 0.001). Considering the MCh challenge, there was a modest effect of time (*F* = 4.034, *P* = 0.047), but a large difference among treatments (*F* = 21.676, *P* < 0.001) in PD20 after 5 years (Fig. 2), again due mostly to the control group that had a lower MCH PD20 at 5 years (*F* = 18.821, *P* <0.001; post hoc tests with Sidak’s correction, *P* < 0.001). No significant difference in PD20 was detected between sSLIT and aSLIT (post hoc test with Sidak’s correction, *P* > 0.050). The FEV1 was significantly decreased in controls, with no change in the sSLIT group and a significant increase in aSLIT as compared to sSLIT (Fig. 3) (post hoc tests with Sidak’s correction, *P* < 0.001).

**Table 1:** Demographic and clinical characteristics of the 124 subjects having the complete data at baseline and at 5 years. Significant *P* values for One way ANOVA are reported; ns: *P* > 0.05

		Control	sSLIT	aSLIT	*P*
Sex	F	17	20	18	ns
	M	23	23	23	
Age	Mean (SEM)	27.08 (1.74)	26.09 (1.83)	24.83 (1.71)	ns
SMS^a^	Mean (SEM)	291.03 (10.81)	283.58 (14.13)	305.78 (10.89)	ns
MCH PD20^b^	Mean (SEM)	1951.98 (35.07)	1958.33 (35.51)	1988.56 (34.75)	ns
FEV1^c^	Mean (SEM)	95 (0.97)	94.44 (1.06)	96.73 (0.81)	ns

^a^Symptom + Medication Score

^b^MCh PD20: provocation dose causing a FEV1 20% decrease

^c^FEV1: Forced expiratory volume in 1 s (% predicted)

**Table 2:** New sensitizations occurred at 5 years subdivided per allergen. The last row reports the number and percentage of patients with at least one new sensitization

	aSLIT *n* = 37	sSLIT *n* = 38	Control *N* = 31
Grass	1	3	8
Birch	2	3	6
Cat	1	3	3
Alternaria	1	1	4
Parietaria	1	-	2
Olive	-	-	1
Ragweed	-	1	2
Dog	1	1	1
Total patients (%)	3 (8.1)	5 (13.2)	18 (58.1)

**Table 3:** Evaluated parameters at 5 years in the 3 groups (mean, standard errors of means, 95% confidence intervals of the mean, and minimum and maximum values)

		Mean	Std. Error	95% Confidence Interval for Mean		Minimum	Maximum
				Lower Bound	Upper Bound		
SMS	Drugs	345.16	19.526	305.28	385.04	121	470
	sSLIT	86.47	14.579	56.93	116.01	13	299
	aSLIT	41.08	13.976	12.74	69.43	7	370
	Total	146.28	15.532	115.49	177.08	7	470
MCh PD20	Drugs	925.48	152.588	613.86	1237.11	68	2380
	sSLIT	1769.84	101.384	1564.42	1975.27	186	2443
	aSLIT	1936.65	75.278	1783.98	2089.32	192	2394
	Total	1581.13	75.241	1431.94	1730.32	68	2443
FEV1	Drugs	87.45	1.585	84.22	90.69	76	106
	sSLIT	95.05	1.172	92.68	97.43	81	113
	aSLIT	104.11	1.268	101.54	106.68	87	117
	Total	95.99	1.000	94.01	97.97	76	117
BETA2	Drugs	5.13	1.226	2.63	7.63	0	19
	sSLIT	.32	.156	.00	.63	0	4
	aSLIT	.16	.099	−.04	.36	0	3
	Total	1.67	.421	.84	2.50	0	19

## Discussion

Main results of this real-life observational study in patients with pure allergic rhinitis due to mite were: a) a reduction of combined symptoms + medication scores of rhinitis, recorded for 4 months at baseline and after 5 years with both the SLIT products was seen; b) the onset of new sensitizations at SPT was significantly lower in the SLIT groups than in the control group; c) there was a significant decrease in MCh PD20 (that is an increase in bronchial hyperreactivity) only in those patients not receiving SLIT; d) a small but significant decrease in FEV1 was seen only in patients treated with symptomatic drugs only; e) the intake of salbutamol, although low, increased in the drug only group after 5 years.

Pending the limitations due to the real-life experimental framework, the information is of clinical relevance, since it confirms that AIT (SLIT in the present case) may affect the onset of asthma and intervene on the lung function. To our knowledge, this is the only study evaluating unselected patients at 5 years after starting AIT using also objective parameters. Considering the real-life setting, that requires the use of standard of care procedures only, no randomization or blinding was made. Also, there were no selection criteria, but the eligibility to receive AIT according to guidelines. This observational study has, therefore, the unavoidable limitations of all studies that are conducted in a real life setting, with standard procedures and standard of care. In such studies, blinding and placebo control are not feasible, and interim parameters are lacking, since they cannot be obtained for all patients. On the other hand, the small number of patients lost to follow-up after 5 years, indirectly testifies for the persistence of subjects with the prescribed care. Also, there was no randomization procedure, and the type of treatment was agreed with patients by physicians in all cases. The strengths reside in the long period of observation, in the fact that objective measures were performed, and that the observations were performed in unselected patients, seen at a territorial Unit.

AIT (either SLIT and SCIT) has been robustly demonstrated clinically effective in reducing symptoms and medication consumption in respiratory allergy [1–3], and the magnitude of this effect has been largely confirmed in the so-called “big trials” conducted with grass, ragweed and mite [6, 14]. According to this, several extracts have been approved as pharmacological products by the European Medicine Agency and the Food and Drug Administration [15]. The majority of trials were conducted in rhinitis and with pollen extracts, and fewer in asthma and with mites [16, 17]. The reason of this, stands in the fact that with mites there are more methodological problems, including the variable exposure and the need for long periods of observation [18]. In addition, asthma is more difficult to assess, since objective measures (PFT) are needed [19]. Nonetheless, consistent data supported the effect of AIT in this disease [16, 17]. Another aspect, linking rhinitis, asthma and AIT is that rhinitis is a strong and independent risk factor for developing asthma [20] and AIT can intervene on this progression [6]. The use of adjuvants in AIT was repeatedly proposed (for review see [21, 22]), and some products containing bacterial adjuvants are already commercialized and used [23]. In general, the addition of an adjuvant allows to reduce the total dose of allergen to be administered, by enhancing the TH1 response via toll like receptors stimulation [24].

In the present observational trial, conducted in real life and using standard of care procedures, we evaluated the effects of SLIT (using two marketed products, one with bacterial adjuvants) on respiratory function in patients with pure allergic rhinitis due to mites at baseline, and we observed a preventative effect on the development of new sensitizations, asthma symptoms and changes in pulmonary function.

## Conclusion

In conclusion, when assessed in a real life setting, both sSLIT and aSLIT showed a preventative effect on pulmonary function changes over a 5-year period, in patients who were initially treated for a pure allergic rhinitis.
